# Dose-response relationship between iTBS and prefrontal activation during executive functioning: A fNIRS study

**DOI:** 10.3389/fpsyt.2022.1049130

**Published:** 2022-12-20

**Authors:** Bella B. B. Zhang, Rebecca L. D. Kan, Cristian G. Giron, Tim T. Z. Lin, Suk-Yu Yau, Georg S. Kranz

**Affiliations:** ^1^Department of Rehabilitation Sciences, The Hong Kong Polytechnic University, Hung Hom, Hong Kong SAR, China; ^2^Mental Health Research Center (MHRC), The Hong Kong Polytechnic University, Hung Hom, Hong Kong SAR, China; ^3^Department of Psychiatry and Psychotherapy, Comprehensive Center for Clinical Neurosciences and Mental Health (C3NMH), Medical University of Vienna, Vienna, Austria; ^4^The State Key Laboratory of Brain and Cognitive Sciences, The University of Hong Kong, Hung Hom, Hong Kong SAR, China

**Keywords:** intermittent theta burst stimulation, stimulation intensity, functional near infrared spectroscopy, dorsolateral prefrontal cortex, executive function

## Abstract

**Introduction:**

Intermittent theta-burst stimulation (iTBS) is a non-invasive brain stimulation paradigm that has demonstrated promising therapeutic benefits for a variety of neuropsychiatric disorders. It has recently garnered widespread favor among researchers and clinicians, owing to its comparable potentiation effects as conventional high-frequency repetitive transcranial magnetic stimulation (rTMS), but administered in a much shorter time frame. However, there is still a lack of agreement over the optimal stimulation intensity, particularly when targeting the prefrontal regions. The objective of this study was to systematically investigate the influence of different stimulation intensities of iTBS, applied over the left dorsolateral prefrontal cortex (DLPFC), on brain activity and executive function in healthy adults.

**Methods:**

Twenty young healthy adults were enrolled in this randomized cross-over experiment. All participants received a single session iTBS over the left DLPFC at intensities of 50, 70, or 100% of their individual resting motor threshold (RMT), each on separate visits. Functional near-infrared spectroscopy (fNIRS) was used to measure changes of hemoglobin concentrations in prefrontal areas during the verbal fluency task (VFT) before and after stimulation.

**Results:**

After stimulation, iTBS to the left DLPFC with 70% RMT maintained the concentration change of oxyhemoglobin (HbO) in the target area during the VFT. In contrast, 50% [*t*_(17)_ = 2.203, *P* = 0.042, *d* = 0.523] and 100% iTBS [*t*_(17)_ = 2.947, *P* = 0.009, *d* = 0.547] significantly decreased change of HbO concentration, indicating an inverse U-shape relationship between stimulation intensity and prefrontal hemodynamic response in healthy young adults. Notably, improved VFT performance was only observed after 70% RMT stimulation [*t*_(17)_ = 2.511, *P* = 0.022, *d* = 0.592]. Moreover, a significant positive correlation was observed between task performance and the difference in HbO concentration change in the targeted area after 70% RMT stimulation (*r* = 0.496, *P* = 0.036) but not after 50 or 100% RMT stimulation.

**Conclusion:**

The linear relationship between stimulation intensity and behavioral outcomes reported in previous conventional rTMS studies may not be translated to iTBS. Instead, iTBS at 70% RMT may be more efficacious than 100% RMT.

## Introduction

Transcranial magnetic stimulation (TMS) is a well-established non-invasive brain stimulation technique that elicits action potentials through application of a magnetic field on the scalp (1). Repetitive transcranial magnetic stimulation (rTMS) has been shown to modify cortical excitability beyond the stimulation session. The underlying mechanism of these effects may be related to modulated long-term potentiation (LTP) and long-term depression (LTD), as observed in animal studies (2). Recently, theta-burst stimulation (TBS), a potent form of rTMS, has gained increased attention, due to its comparable potentiation effects as conventional rTMS, but administered in a much shorter time frame (3). TBS consists of a series of 3-pulse bursts at 50 Hz (theta rhythm), designed to mimic the firing patterns of hippocampal neurons in rats (4) and has been demonstrated to optimally induce LTP in animal studies (5). In humans, TBS protocols were first tested on the primary motor cortex at an intensity of 80% active motor threshold (AMT) by Huang et al. (3) who showed that the intermittent form of TBS (iTBS) induces excitatory effects while the continuous form of TBS (cTBS) induces inhibitory effects on brain activity. Since its first description, TBS has been applied to other non-motor areas. The past decade has seen the rapid development of application of TBS on the dorsolateral prefrontal cortex (DLPFC) for the treatment of various neurological and psychiatric disorders (6–9). However, questions have been raised about the optimal parameters for maximizing the response to TBS. For instance, one of the current discussions pertains to the TBS intensity used for the DLPFC neuromodulation. Huang and Rothwell (10) reported an increased MEP with increasing intensity (50, 70, and 80% AMT) of 50 Hz burst stimulations of the motor cortex (10). In more recent treatment studies, TBS is used at wide ranging intensities, from 80% AMT to 120% resting motor threshold (RMT) (11, 12). In conventional rTMS studies, an almost linear relationship between stimulation intensity and neuromodulation is assumed in conventional rTMS studies (13, 14). However, caution should be taken when directly transferring this relationship from conventional rTMS to TBS, as the mechanism by which they alter brain excitability appears to differ (2, 15, 16). Furthermore, it is still unknown whether the linear relationship reported by Huang and Pothwell using low (50–80% AMT) TBS intensity in motor cortex also exists in high (≧80% AMT) intensity prefrontal stimulation.

Functional near infrared spectroscopy (fNIRS) allows to assess the concentration change of oxygenated hemoglobin (HbO) and deoxygenated hemoglobin (HbR) in biological tissue (17). This is achieved by transmitting near infrared light (∼700–1,000 nm) into the brain and taking advantage of the transparency difference of tissue within this near infrared optical window (18). fNIRS has been demonstrated to be a very promising tool to monitor functional brain activity in a wide range of applications and populations, especially for the frontal lobe (17, 19). Previous studies reported a robust correlation between the NIRS signal, and the blood oxygenation level dependent (BOLD) signal as measured by functional magnetic resonance imaging (fMRI) (20–22). In the past three decades, fNIRS has become increasingly popular due to its low cost, safety, portability, and tolerability (19, 23, 24). The verbal fluency task (VFT) is a widely used neuropsychological test to evaluate executive functions in which subjects are instructed to generate as many unique words as possible from a category (phonemic or semantic) within a given time limit (25, 26). Previous and recent research demonstrate VFT-induced activation in frontal cortices, including the left DLPFC (27, 28).

Our study set out to systematically investigate the influence of different stimulation intensities of iTBS, applied at the left DLPFC, on brain activity and executive function in healthy adults. We probed (1) a hypothesized linear relationship between iTBS intensity and activation of the DLPFC; and (2) a linear relationship between task performance and stimulation intensity.

## Materials and methods

### Participants

Convenience sampling was used for recruitment at the Hong Kong Polytechnic University from May 2021 to July 2021. We included 20 right-handed, healthy adults in this study (age: 22.3 ± 3.54 years, 10 female). Participants had to be native Chinese speakers between the age of 18 and 35 years and completed at least 6 years of formal education. They had to have normal or corrected to normal eyesight and be able to understand the verbal instructions. Subjects with any of the following conditions were excluded from this study: (1) a history of seizure; (2) current or past psychiatric disorders; (3) current or past severe internal or neurological illness; (4) any TMS contraindications; (5) history of substance dependence or abuse within the last 3 months; (6) intake of any medication (i.e., benzodiazepines, anticonvulsants) known to affect the excitation threshold. The study was conducted according to the Declaration of Helsinki and received the ethical approval from the Human Subjects Ethics Subcommittee (HSESC20181212008) of the Hong Kong Polytechnic University. Written informed consent was obtained from all participants before enrollment.

### Study design and setting

**FIGURE 1:**
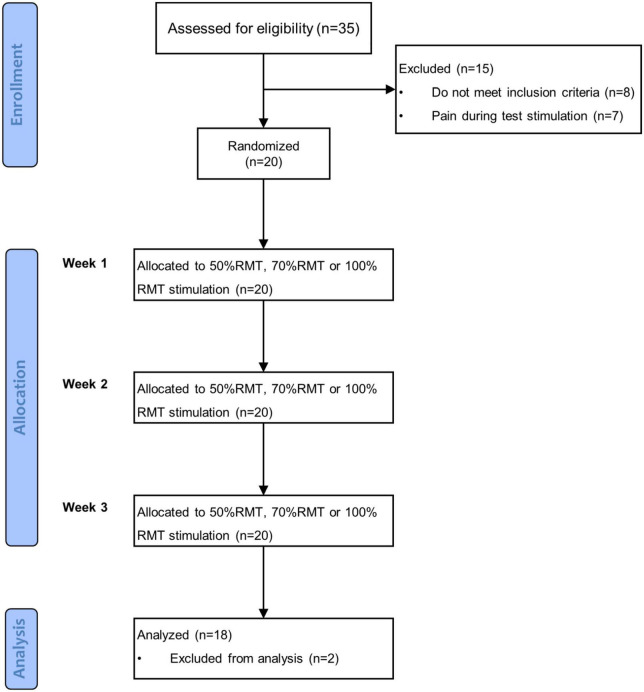
Flowchart of the experiment procedure.

This study was a prospective, randomized cross-over clinical trial with repeated measures. Subjects were instructed to visit our lab three times with 7–9 days between each visit. After enrollment, they were randomly assigned to receive iTBS at an intensity of 50, 70, or 100% RMT in each session. The sequence of stimulation intensities was determined by a simple, computer-generated, random number list, and counterbalanced among subjects. fNIRS measurements were performed immediately before and around 15 min (i.e., the time required to place the fNIRS probe on a subject’s head) after stimulation. During both fNIRS measurements, before and after stimulation, subjects performed the VFT. The summary of the procedure is illustrated in the flowchart shown in Figure 1. This study is a part of a research program which has been registered at clinicaltrials.gov (NCT04031105).

### Intermittent theta burst stimulation

iTBS was delivered using a figure-of-eight shaped cooling coil (Cool-B65), connected to a MagPro magnetic stimulator (MagVenture, Denmark). We adhered to the initial 3-min iTBS protocol (3 pulses × 10 bursts × 20 trains = 600 pulses) developed by Huang et al. (3), which consists of 20 trains of 3-pulse bursts with 50 Hz intra-burst frequency. Each train contains 10 bursts delivered at 5 Hz and separated by 8 s of rest. The RMT for each subject was determined using a single pulse at the left primary motor cortex, defined as the minimum intensity capable of eliciting motor evoked potentials (MEPs) with at least 50 μV peak-to-peak amplitude in at least five out of 10 consecutive measurements of the relaxed right first dorsal interosseous muscle (FDI). iTBS was delivered at intensities of 50, 70, or 100% RMT for each subject on a given session. 70% RMT was chosen because it corresponds to 80% AMT used in study that first reported the application of TBS in human (3). We utilized 100% RMT due to the higher neural activity response to increased stimulation intensity observed in conventional rTMS studies (13, 29). In addition, stimulation at 50% RMT was regarded as an active control condition as a previous study reported that TBS at a low intensity did not affect brain excitability (30). We targeted the left DLPFC at the MNI coordinate of (x-38, y + 44, z + 26), as done previously (31, 32). The stimulation target was identified and monitored by a navigation system (LOCALITE^®^ TMS Navigator Germany) during iTBS. Self-reported side effects were documented after each stimulation, using the self-rate Numeric Pain Rating Scale [from 0 (No Pain) to 10 (Worst Imaginable Pain)] (33). All subjects were naïve to TMS.

### fNIRS measurement

**FIGURE 2:**
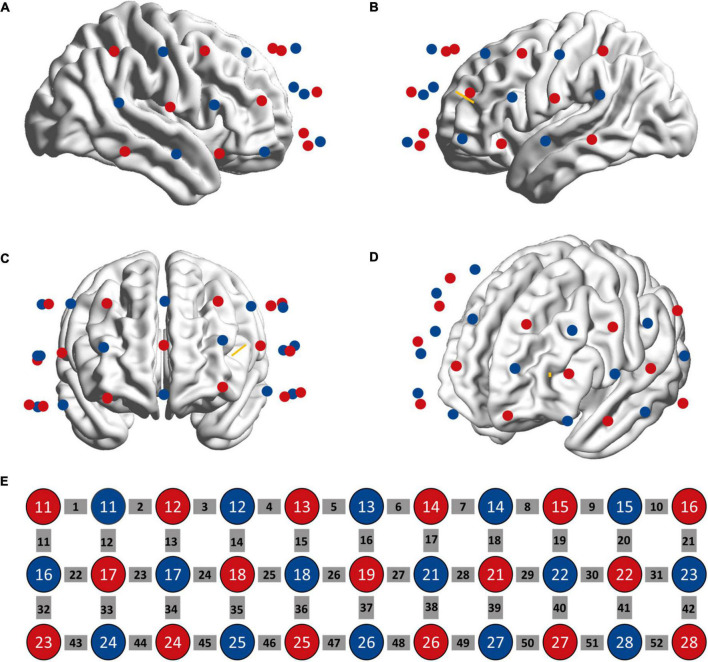
Location of fNIRS sources (red circle) and detectors (blue circle) on the head as seen from the right **(A)**, left **(B)**, anterior **(C)**, and left frontal **(D)** perspectives. **(E)** The sources and detectors were laid out in a 3*11 configuration. The gray squares represent measuring channels. The yellow bar denotes the projection of stimulation target.

Hemodynamic activity was measured using a continuous wave near-infrared (695 and 830 nm) spectroscopy device (ETG-4000, Hitachi Medical Co., Tokyo, Japan) with a sampling rate of 10 Hz. We used a 3 × 11 probe design with 52 channels for data collection (Figure 2). The probe was placed on the forehead with the lower edge aligned with the T4-Fpz-T3 line of International 10–20 system and the sixth column aligned with the brain’s middle line. The area between two nearby sources and detectors is defined as a channel (Ch). The distance between a pair of emitter and detector was 3 cm, which allowed to measure the concentration change of HbO and HbR at 2–3 cm below the skin and scalp surface. The probe was registered to the surface of the standard brain embed in the AtlasViewer toolbox (34) and projected to the cortex to estimate the MNI coordinate of each channel (the midpoint between each pairs of source and detector). The estimation of probabilistic anatomical locations of channels based on the Brodmann area (BA) atlas shows that our probe arrangement enabled to detect the hemoglobin changes in bilateral DLPFC (BA 9, 46), frontopolar area (BA 10), anterior superior temporal gyrus (BA 22), and middle temporal gyrus (BA 21). MNI coordinates of each channel’s midpoint and the estimated corresponding BA area for each channel are shown in Supplementary material. Participants were told to sit still and avoid head movements during the measurement. Measurements started once the fNIRS signal was stable.

### Verbal fluency task

The design of the VFT was adapted from previous fNIRS studies, which utilized a counterbalanced block design (26, 35, 36). The task consisted of two experimental blocks and two control blocks. Animals and means of transportation were used as semantic categories for the VFT before stimulation (VFT version 1) while clothes and fruits/vegetables were used as categories for the VFT after stimulation (VFT version 2). Both versions started with a 60-s block of the control condition, followed by a 60-s block of the experimental condition. During the experiment, subjects sat comfortably and 50 cm in front of a computer screen. During the experimental condition, participants were told to generate as many words as possible that belonged to the semantic category shown on the center of the screen without repetition. During the control block, they needed to repeat the numbers “1, 2, 3, 4, 1, 2, 3, 4…” at a steady pace, as done previously (26). The purpose of these control blocks was to account for changes in hemodynamic response caused by talking. Prior to the start of the task, subjects were given a practice trial (i.e., semantic category of flowers) to ensure that they understood how to complete the task correctly. The overall duration of the VFT task was 240 s. All stimuli were presented using E-Prime 2 (Psychology Software Tool, Pittsburgh, PA, USA).

### Data analysis

#### fNIRS data analysis

fNIRS data analysis was performed using the HOMER 2 toolbox and custom scripts developed in MATLAB 2013b (The MathWorks, Inc., Natick, MA) (37). For preprocessing, channels with an optical density higher than 140 dB were excluded for further analysis to omit saturated channels (38). The raw fNIRS data was then converted to optical density (OD) (39). Motion artifacts were detected and corrected by a mixed approach based on the spline interpolation method and Slvitzky-Golay filtering of each channel (40). A bandpass filter (0.002–0.08 Hz) was then employed to remove physiological noise caused by heartbeat, respiration, and drifts (41). These preprocessed signals were converted to the concentration change of HbO (ΔHbO) based on the modified Beer-Lambert Law with a differential pathlength factor of six (42, 43). The time course HbO concentration change (0–60 s) for the experimental conditions was calculated using the *hmrR_BlockAvg* function (37). Lastly, the data of experimental blocks were averaged. Baseline correction was performed per experimental block using the mean of the last 5 s signal of control block (44). We also investigated the hemodynamic response during the early VFT task period (0–30 s) because a previous study reported that the early semantic VFT phase (0–30 s) was supported by executive functions while the late phase (31–60 s) was mainly dependent on the semantic network activation (45). The region of interest was defined as the stimulated area that corresponds most closely to the location of Ch28. In this study, we used HbO signals as an indicator of hemodynamic response since HbO is more sensitive to regional cerebral blood flow than Hb (46). The mean of HbO concentration change for different groups were used for further analysis. To visualize the difference of the left DLPFC activation during VFT task before and after stimulation, we contrasted the mean of ΔHbO (0–60 s) before and after stimulation for each intensity using paired *t*-test results. This analysis yielded three t-maps which show the *t*-value for ch28 at each intensity. The *t*-values and MNI coordinates were first converted to *.img files using nirs2im function^1^ in the xjview toolbox.^2^ Next, the transformed image files were visualized on a 3D brain model (ICBM512 template) using a BrainNet Viewer toolbox (47).

#### Statistical analysis

One-way repeated measures analysis of variance (ANOVA) was used to compare the baseline difference of VFT performance and brain activity. In order to investigate the stimulation effects, behavioral as well as imaging data were analyzed by two-way repeated measures ANOVA using time (pre, post) and intensity (50, 70, and 100%) as within-subjects factors. In case of significant main effects, *post* hoc pairwise comparisons were corrected using Fisher least significant difference (LSD) procedure in accordance with the closed test principle: *post* hoc comparisons were declared non-significant if the global *p*-value of the main effect (testing equality of both time points or of all 3 intensities simultaneously) was non-significant but carried out without further correction in case of a significant global main effect. Statistical significance was set at *P* < 0.05. Pearson correlation was used to analyze the relationship between VFT performance and difference of ΔHbO. SPSS version 24 for Windows (SPSS Inc., Chicago, IL)^3^ was used for statistical analyses.

## Results

**FIGURE 3:**
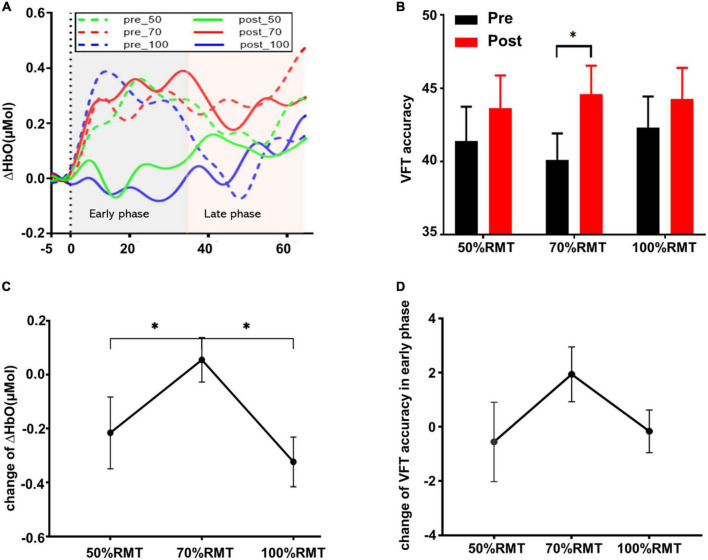
**(A)** Group averaged time course HbO concentration change during the experimental condition in the left DLPFC (Ch28) before and after stimulation at each intensity. The *Y*-axis represents the mean of ΔHbO. **(B)** VFT behavior performance (mean ± SEM) for the whole task period (0–60 s). **(C)** Change of ΔHbO in the early task phase (0–30 s) for each stimulation intensity. Data were calculated by subtracting the mean of ΔHbO before stimulation from the mean of ΔHbO after stimulation. **(D)** VFT behavior performance change (mean ± SEM) in early task phase for each stimulation intensity. *p < 0.05.

**FIGURE 4:**
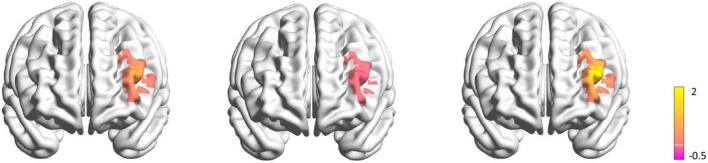
Maps of the left DLPFC activation difference before and after stimulation during VFT task in 50% RMT condition **(left)**, 70% RMT condition **(middle)**, 100% RMT condition **(right)**. The color bar indicates the *t*-values render over on a 3D head model. The yellow color represents less ΔHbO after stimulation, while the purple color represents more ΔHbO after stimulation.

**FIGURE 5:**
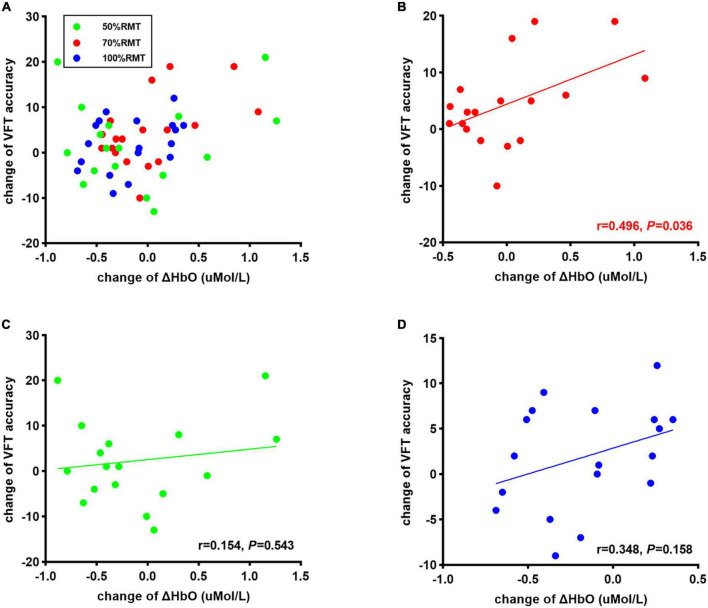
**(A)** Correlation between change of corrected words generated during VFT task and change of ΔHbO at all stimulation intensities Correlation between change of corrected words generated during VFT task and change of ΔHbO in 70% RMT stimulation **(B)**, 50% RMT stimulation condition **(C)**, and 100% RMT stimulation **(D)**.

Two subjects were excluded from the data analysis due to poor quality of fNIRS signals and interruption of the program during measurement. Finally, 18 subjects (9 female, mean age: 22.30 ± 3.54 years) were included for the data analysis. One-way repeated measures ANOVA did not reveal a significant difference in the time interval between the second fNIRS measurement and iTBS between groups [50%RMT group: 20.780 ± 2.533 min; 70%RMT group: 19.889 ± 2.720 min; 100%RMT group: 20.778 ± 2.942 min; *F*_(2_, _34)_ = 0.727, *P* = 0.491]. Prior to stimulation, the subjects generated 41.39 ± 10.05, 40.11 ± 7.75, 42.33 ± 9.00 accurate words for 50, 70, and 100% RMT stimulation condition, respectively. After stimulation these values increased to 43.67 ± 9.42 [*t*_(17)_ = 1.06, *P* = 0.305, *d* = 0.249], 44.61 ± 8.24 [*t*_(17)_ = 2.511, *P* = 0.022, *d* = 0.592], and 44.28 ± 9.04 [*t*_(17)_ = 1.421, *P* = 0.173, *d* = 0.335], respectively. The averaged fNIRS signal during the VFT task in the left DLPFC (Ch28) among different conditions is shown in Figure 3A. There were no baseline differences regarding VFT behavioral performance [*F*_(2_, _34)_ = 0.500, *P* = 0.611, η*_*p*_*^2^ = 0.029] and brain activity [*F*_(2_, _34)_ = 0.267, *P* = 0.767, η*_*p*_*^2^ = 0.015] between groups. Two-way repeated measures ANOVA on VFT accuracy showed a significant main effect of time [*F*_(1_, _17)_ = 4.455, *P* = 0.05, η*_*p*_*^2^ = 0.208] but not of intensity [F_(2_, _34)_ = 0.165, *P* = 0.849, η*_*p*_*^2^ = 0.010] nor an interaction of time × intensity [*F*_(2_, _34)_ = 0.958, *P* = 0.394, η*_*p*_*^2^ = 0.053]. Exploratory *post* hoc comparisons indicated a significant performance increase after stimulation compared to baseline at the intensity of 70%RMT [*t*_(17)_ = 2.511, *P* = 0.022, *d* = 0.592] but not at the other two intensities (*P* > 0.05) (Figure 3B). Brain activation analysis for the early task period showed a significant main effect of time [*F*_(1_, _17)_ = 4.873, *P* = 0.041, η*_*p*_*^2^ = 0.223], and an interaction effect of time and intensity [*F*_(2_, _34)_ = 4.442, *P* = 0.019, η*_*p*_*^2^ = 0.207] but no main effect of intensity [*F*_(2_, _34)_ = 2.130, *P* = 0.134, η*_*p*_*^2^ = 0.111]. *Post* hoc analyses using change scores to resolve the interaction effect indicated significantly higher HbO values post stimulation at 70% RMT, compared to 50% [*t*_(17)_ = 2.203, *P* = 0.042, *d* = 0.523] and 100% RMT [*t*_(17)_ = 2.947, *P* = 0.009, *d* = 0.547] (Figure 3C). Analysis for early phase behavioral performance also showed the same inverse U-shape curve despite not reaching significance [time: *F*_(1_, _17)_ = 0.349, *P* = 0.563, η*_*p*_*^2^ = 0.020; intensity: *F*_(2_, _34)_ = 0.015, *P* = 0.985, η*_*p*_*^2^ = 0.001; time × intensity: *F*_(2_, _34)_ = 1.528, *P* = 0.232, η*_*p*_*^2^ = 0.082] (Figure 3D). No significant results were observed when looking at the HbO change averaged across the whole task period (Figure 4). However, correlation analysis revealed a significant positive correlation between behavioral accuracy and the difference in HbO concentration change in left DLPFC after 70% RMT stimulation (Pearson’s *r* = 0.496, *P* = 0.036) but not after the other two intensities (Figure 5).

## Discussion

In this study, we investigated the effects of varying stimulation intensities of iTBS of the left DLPFC on executive function and underlying cortical activity using fNIRS. While several previous studies have investigated iTBS effects on the brain using EEG (9, 48, 49), we investigated such effects using fNIRS, as it is more tolerant to lip and jaw movements during VFT task performance (19). In all stimulation intensity, descriptively, the number of words generated by subjects after stimulation was increased than before stimulation. However, the improvement of executive performance was only significant after 70% RMT stimulation. Besides, iTBS to the left DLPFC with 70% RMT maintained the concentration change of HbO in the target area, whereas 50% iTBS and 100% iTBS decreased change of HbO concentration, indicating an inverse U-shape relationship between stimulation intensity and prefrontal hemodynamic response. Moreover, a significant positive correlation was observed between behavioral accuracy and the difference in HbO concentration change in the targeted area after 70% RMT stimulation.

The modest enhancements of VFT performance in all intensity conditions supports the beneficial effects of excitatory stimulation of iTBS stimulation on executive functioning (50, 51). However, contrary to our hypothesis, our results did not demonstrate a linear relationship between stimulation intensity and brain activity, as observed in conventional rTMS studies (13, 52) and a study examining low TBS intensity (10). Specifically, we found significant improvement of executive function only after 70% RMT iTBS; the corresponding hemodynamic response revealed that iTBS to the left DLPFC with 70% RMT maintained the concentration change of HbO in the target area, whereas 50% iTBS and 100% iTBS decreased ΔHbO. A possible explanation for these observations is related to mechanisms of theta-frequency-dependent LTP induction (53, 54). Normally, a single burst activates a glutamatergic (excitatory) synapse and also a gamma-aminobutyric acid (GABA)-ergic (inhibitory) synapse on a pyramidal neuron, producing both an excitatory postsynaptic potential (EPSP) and inhibitory postsynaptic potential (IPSP) on this neuron. This IPSP undercuts the EPSP triggered by this burst and the next burst for a short period of time, preventing the hyperexcitability of the downstream pyramidal neuron. However, this IPSP can be suppressed for a short period of time if a second burst is delivered at theta frequency (53). The underlying mechanism is further GABA release from the GABAergic presynaptic terminal that follows this second burst, inhibits future GABA release through activation of GABA_B_ autoreceptors (55). Consequently, this GABA-mediated disinhibition induces LTP effects via activation of N-methyl-D-aspartate (NMDA) receptors (56). A previous study reported that a second burst delivered at 200 ms after the initial burst produces maximal excitatory effects by using this mechanism of disinhibition, i.e., by inducing more GABA release to activate GABA_B_ autoreceptors (54, 55). Bursts given outside of this window (above or below 200 ms) may not result in such optimal effects in human brains, possibly because burst effects encounter an already-present IPSP from previous activations or recovered IPSP (53, 57). An important caveat it that the onset of this disinhibition may be modulated by stimulus intensity (57). Therefore, TBS at high intensities (such as 100% RMT) may off-set this temporal window and fail to elicit the maximal excitatory effects of theta frequency on brain activity. Consistent with this view, Chung et al. found that iTBS at 75% RMT intensity showed maximal neuromodulatory effects on brain activity in humans (58).

We observed a relative lower ΔHbO following 50 and 100% RMT stimulation compared to before stimulation despite increased VFT performance. This appears to be contradictory to the general understanding of the neurovascular coupling phenomenon. According to this principle, a cognitively demanding task such as the VFT should lead to a rise in HbO needs, indicating an increase in cortical activation, as a result of increased neuronal mobilization (28, 59, 60). It is theorized that higher cortical activation should be accompanied with a better behavioral performance, since higher cortical activation suggests more cognitive resources are being mobilized to complete a task (61). However, previous studies also reported that increased DLPFC activation may be a compensatory strategy for reduced available neural resources, or alternatively, an inefficient employment of neural resources (62, 63). Recent evidence suggests that excitatory rTMS to the left DLPFC increases neural efficiency, observed as reduced concentration change of total hemoglobin after stimulation during Speed of Processing task (64). This finding corroborates cognitive efficiency theories which propose that people with a more efficient cortical processing require less cognitive resources to achieve better performance (62, 65, 66). Therefore, TBS benefits to behavioral performance may be due to improved efficiency of neurons, such that the same levels of cortical activation (captured by fNIRS) provides increased processing power, improving performance.

Imaging results revealed a significantly higher HbO concentration change following 70% RMT stimulation than 50% RMT and 100% RMT only in the early task phase but not the whole task period. This can be explained by the dynamic model of retrieval process involved in the semantic fluency tasks. A previous study indicated that early phases of the semantic VFT task is mediated more by executive processes while the late phase is mainly dependent on semantic network activation (45). It has been well established that the DLPFC plays an important role in supporting executive control (67–70). Therefore, it is not surprising that the potentially optimal iTBS intensity enhanced the excitability of the left DLPFC and further boosted the behavioral performance.

Certainly, our study is not free of limitations. First, we did not have a real sham condition in this experiment. Nonetheless, our study included a low intensity (50% RMT) condition, comparable to a number of TMS studies that have adopted the strategy of lowering stimulation intensity as a sham condition (49, 71, 72). Second, due to the inherent limitation of the fNIRS equipment used, such as the height profile of our fNIRS probes we were unable to measure the hemodynamic response to iTBS during and immediately after the stimulation. To demonstrate this, further studies using a concurrent TMS-fNIRS set up are needed. Thirdly, Fisher LSD method does not offer full control of the type I error. However, it is known to preserve the experiment-wise type I error at the nominal significance level if there are three groups (73). Fourth, the choice of intensities used in our study is not representative of all often-used stimulation intensities in clinical settings (e.g., 90, 110, and 120% RMT). We adopted relatively lower TBS intensities, as an endorsed advantage of TBS protocols in clinical applications is the lower necessary intensity for treatment, allowing for more comfortable sessions (74, 75). Additionally, on methodological grounds, our results are comparable to Huang et al. (3), who used low intensities (80% AMT) to study the patterned effects of TBS on MEPs. Even so, our findings have limited generalizability to suprathreshold TBS intensities. Further studies comparing these effects are needed.

## Conclusion

The linear association between stimulation intensity and behavioral improvement observed in healthy people receiving conventional rTMS may not extend to iTBS. Our investigation revealed an inverted U-shaped association between iTBS intensity and the excitatory effects on brain activity, suggesting that iTBS at 70% RMT may be more efficacious than 100% RMT.

## Data availability statement

The raw data supporting the conclusions of this article will be made available by the authors, without undue reservation.

## Ethics statement

The studies involving human participants were reviewed and approved by the Human Subjects Ethics Subcommittee of the Hong Kong Polytechnic University. The patients/participants provided their written informed consent to participate in this study.

## Author contributions

BZ: conceptualization, methodology, software, investigation, data curation, formal analysis, writing—original draft, writing—review and editing, and visualization. RK: methodology, software, formal analysis, writing—review and editing. CG: methodology, formal analysis, and writing—review and editing. TL: software, formal analysis, visualization, and writing—review and editing. S-YY: writing—review and editing and supervision. GK: resources, conceptualization, methodology, writing—original draft, writing—review and editing, supervision, project administration, and funding acquisition. All authors contributed to the article and approved the submitted version.

## Footnotes

1
https://www.alivelearn.net/?p=2230

2
https://www.alivelearn.net/xjview/

3
www.spss.com
