# Supplementary material for: Dose-response relationship between iTBS and prefrontal activation during executive functioning: A fNIRS study
Source: Front Psychiatry. 2022 Dec 20;13:1049130. doi: 10.3389/fpsyt.2022.1049130 (PMC9807664; doi:10.3389/fpsyt.2022.1049130)
Supplement: Supplementary file 1 [file Table_1.docx]

**Dose-response relationship between iTBS and prefrontal activation during executive functioning: A fNIRS study**

Bella B.B. ZHANG^a^, Rebecca L.D. KAN^a^, Cristian G. GIRON^a^, Tim T.Z. LIN^a^,
Suk-Yu YAU^ab^, Georg S. KRANZ^a,b,c,d^

^a^ Department of Rehabilitation Sciences, The Hong Kong Polytechnic University, Hong Kong SAR, China

^b^ Mental Health Research Center (MHRC), The Hong Kong Polytechnic University, Hong Kong SAR, China

^c^ Department of Psychiatry and Psychotherapy, Comprehensive Center for Clinical Neurosciences and Mental Health (C3NMH), Medical University of Vienna, Vienna, Austria

^d^ The State Key Laboratory of Brain and Cognitive Sciences, The University of Hong Kong, Hong Kong SAR, China

**This file includes:**

Table S1

Table S1. MNI coordinates of each channel’s midpoint and the estimated corresponding BA area for each channel.

| **Channel number** | **MNI coordinate** | | | **Brodmann area** | **Estimated probability** |
| --- | --- | --- | --- | --- | --- |
|  | **X** | **Y** | **Z** |  |  |
| CH01 | 62.333 | -42.333 | 49.667 | 40 - Supramarginal gyrus part of Wernicke's area | 1 |
|  |  |  |  |  |  |
| CH02 | 60.333 | -13.667 | 50.667 | 4 - Primary Motor Cortex | 0.394 |
|  |  |  |  | 3 - Primary Somatosensory Cortex | 0.344 |
|  |  |  |  | 6 - Pre-Motor and Supplementary Motor Cortex | 0.151 |
|  |  |  |  | 1 - Primary Somatosensory Cortex | 0.112 |
|  |  |  |  |  |  |
| CH03 | 50 | 13.333 | 51.333 | 9 - Dorsolateral prefrontal cortex | 0.612 |
|  |  |  |  | 6 - Pre-Motor and Supplementary Motor Cortex | 0.376 |
|  |  |  |  | 44 - pars opercularis, part of Broca's area | 0.012 |
|  |  |  |  |  |  |
| CH04 | 31.333 | 34.333 | 52.333 | 9 - Dorsolateral prefrontal cortex | 0.642 |
|  |  |  |  | 8 - Includes Frontal eye fields | 0.358 |
|  |  |  |  |  |  |
| CH05 | 9 | 47 | 53 | 9 - Dorsolateral prefrontal cortex | 0.687 |
|  |  |  |  | 8 - Includes Frontal eye fields | 0.313 |
|  |  |  |  |  |  |
| CH06 | -15.667 | 45.667 | 50.333 | 9 - Dorsolateral prefrontal cortex | 0.893 |
|  |  |  |  | 8 - Includes Frontal eye fields | 0.107 |
|  |  |  |  |  |  |
| CH07 | -37.667 | 31.667 | 48 | 9 - Dorsolateral prefrontal cortex | 0.919 |
|  |  |  |  | 8 - Includes Frontal eye fields | 0.045 |
|  |  |  |  | 46 - Dorsolateral prefrontal cortex | 0.022 |
|  |  |  |  | 45 - pars triangularis Broca's area | 0.009 |
|  |  |  |  | 44 - pars opercularis, part of Broca's area | 0.004 |
|  |  |  |  |  |  |
| CH08 | -51 | 11.667 | 46.667 | 6 - Pre-Motor and Supplementary Motor Cortex | 0.457 |
|  |  |  |  | 9 - Dorsolateral prefrontal cortex | 0.358 |
|  |  |  |  | 44 - pars opercularis, part of Broca's area | 0.185 |
|  |  |  |  |  |  |
| CH09 | -60.333 | -13.667 | 46.667 | 3 - Primary Somatosensory Cortex | 0.357 |
|  |  |  |  | 4 - Primary Motor Cortex | 0.307 |
|  |  |  |  | 1 - Primary Somatosensory Cortex | 0.213 |
|  |  |  |  | 6 - Pre-Motor and Supplementary Motor Cortex | 0.087 |
|  |  |  |  | 43 - Subcentral area | 0.036 |
|  |  |  |  |  |  |
| CH10 | -62.667 | -35.667 | 48.333 | 40 - Supramarginal gyrus part of Wernicke's area | 0.806 |
|  |  |  |  | 2 - Primary Somatosensory Cortex | 0.162 |
|  |  |  |  | 1 - Primary Somatosensory Cortex | 0.032 |
|  |  |  |  |  |  |
| CH11 | 64.333 | -53.333 | 33 | 40 - Supramarginal gyrus part of Wernicke's area | 0.413 |
|  |  |  |  | 22 - Superior Temporal Gyrus | 0.302 |
|  |  |  |  | 39 - Angular gyrus, part of Wernicke's area | 0.252 |
|  |  |  |  | 48 - Retrosubicular area | 0.034 |
|  |  |  |  |  |  |
| CH12 | 69.667 | -23 | 34.333 | 2 - Primary Somatosensory Cortex | 0.731 |
|  |  |  |  | 1 - Primary Somatosensory Cortex | 0.215 |
|  |  |  |  | 40 - Supramarginal gyrus part of Wernicke's area | 0.035 |
|  |  |  |  | 43 - Subcentral area | 0.019 |
|  |  |  |  |  |  |
| CH13 | 63 | 4.667 | 36.667 | 6 - Pre-Motor and Supplementary Motor Cortex | 0.674 |
|  |  |  |  | 4 - Primary Motor Cortex | 0.165 |
|  |  |  |  | 43 - Subcentral area | 0.106 |
|  |  |  |  | 44 - pars opercularis, part of Broca's area | 0.051 |
|  |  |  |  | 3 - Primary Somatosensory Cortex | 0.004 |
|  |  |  |  |  |  |
| CH14 | 48.667 | 32.667 | 38.667 | 45 - pars triangularis Broca's area | 0.421 |
|  |  |  |  | 44 - pars opercularis, part of Broca's area | 0.237 |
|  |  |  |  | 9 - Dorsolateral prefrontal cortex | 0.204 |
|  |  |  |  | 46 - Dorsolateral prefrontal cortex | 0.138 |
|  |  |  |  |  |  |
| CH15 | 24 | 52 | 42.667 | 9 - Dorsolateral prefrontal cortex | 0.955 |
|  |  |  |  | 46 - Dorsolateral prefrontal cortex | 0.045 |
|  |  |  |  |  |  |
| CH16 | -5 | 58.667 | 41 | 9 - Dorsolateral prefrontal cortex | 0.85 |
|  |  |  |  | 10 - Frontopolar area | 0.15 |
|  |  |  |  |  |  |
| CH17 | -30.333 | 48.333 | 38.333 | 9 - Dorsolateral prefrontal cortex | 0.583 |
|  |  |  |  | 46 - Dorsolateral prefrontal cortex | 0.417 |
|  |  |  |  |  |  |
| CH18 | -48.667 | 29.667 | 37.333 | 45 - pars triangularis Broca's area | 0.474 |
|  |  |  |  | 44 - pars opercularis, part of Broca's area | 0.386 |
|  |  |  |  | 9 - Dorsolateral prefrontal cortex | 0.096 |
|  |  |  |  | 46 - Dorsolateral prefrontal cortex | 0.044 |
|  |  |  |  |  |  |
| CH19 | -61 | 5 | 34.667 | 6 - Pre-Motor and Supplementary Motor Cortex | 0.646 |
|  |  |  |  | 43 - Subcentral area | 0.139 |
|  |  |  |  | 4 - Primary Motor Cortex | 0.131 |
|  |  |  |  | 44 - pars opercularis, part of Broca's area | 0.077 |
|  |  |  |  | 3 - Primary Somatosensory Cortex | 0.007 |
|  |  |  |  |  |  |
| CH20 | -66 | -19.333 | 36.667 | 2 - Primary Somatosensory Cortex | 0.492 |
|  |  |  |  | 1 - Primary Somatosensory Cortex | 0.354 |
|  |  |  |  | 43 - Subcentral area | 0.131 |
|  |  |  |  | 3 - Primary Somatosensory Cortex | 0.023 |
|  |  |  |  |  |  |
| CH21 | -65.333 | -45 | 36.333 | 40 - Supramarginal gyrus part of Wernicke's area | 0.817 |
|  |  |  |  | 48 - Retrosubicular area | 0.157 |
|  |  |  |  | 22 - Superior Temporal Gyrus | 0.023 |
|  |  |  |  | 39 - Angular gyrus, part of Wernicke's area | 0.003 |
|  |  |  |  |  |  |
| CH22 | 71 | -35.667 | 17.667 | 22 - Superior Temporal Gyrus | 0.779 |
|  |  |  |  | 48 - Retrosubicular area | 0.118 |
|  |  |  |  | 40 - Supramarginal gyrus part of Wernicke's area | 0.078 |
|  |  |  |  | 2 - Primary Somatosensory Cortex | 0.025 |
|  |  |  |  |  |  |
| CH23 | 69 | -4 | 20 | 43 - Subcentral area | 0.777 |
|  |  |  |  | 22 - Superior Temporal Gyrus | 0.125 |
|  |  |  |  | 6 - Pre-Motor and Supplementary Motor Cortex | 0.05 |
|  |  |  |  | 48 - Retrosubicular area | 0.047 |
|  |  |  |  |  |  |
| CH24 | 60 | 24.667 | 22.667 | 45 - pars triangularis Broca's area | 0.617 |
|  |  |  |  | 44 - pars opercularis, part of Broca's area | 0.383 |
|  |  |  |  |  |  |
| CH25 | 41.667 | 50.667 | 28.667 | 46 - Dorsolateral prefrontal cortex | 0.756 |
|  |  |  |  | 45 - pars triangularis Broca's area | 0.244 |
|  |  |  |  |  |  |
| CH26 | 12.667 | 66 | 30.667 | 10 - Frontopolar area | 0.795 |
|  |  |  |  | 9 - Dorsolateral prefrontal cortex | 0.205 |
|  |  |  |  |  |  |
| CH27 | -19.333 | 62.667 | 29.667 | 10 - Frontopolar area | 0.563 |
|  |  |  |  | 9 - Dorsolateral prefrontal cortex | 0.219 |
|  |  |  |  | 46 - Dorsolateral prefrontal cortex | 0.219 |
|  |  |  |  |  |  |
| CH28 | -42.667 | 46.667 | 27.667 | 46 - Dorsolateral prefrontal cortex | 0.523 |
|  |  |  |  | 45 - pars triangularis Broca's area | 0.477 |
|  |  |  |  |  |  |
| CH29 | -56 | 25 | 25.667 | 45 - pars triangularis Broca's area | 0.592 |
|  |  |  |  | 44 - pars opercularis, part of Broca's area | 0.408 |
|  |  |  |  |  |  |
| CH30 | -65 | -1.333 | 25.667 | 43 - Subcentral area | 0.746 |
|  |  |  |  | 6 - Pre-Motor and Supplementary Motor Cortex | 0.228 |
|  |  |  |  | 4 - Primary Motor Cortex | 0.026 |
|  |  |  |  |  |  |
| CH31 | -69 | -29.333 | 23.667 | 2 - Primary Somatosensory Cortex | 0.419 |
|  |  |  |  | 22 - Superior Temporal Gyrus | 0.309 |
|  |  |  |  | 48 - Retrosubicular area | 0.165 |
|  |  |  |  | 42 - Primary and Auditory Association Cortex | 0.061 |
|  |  |  |  | 40 - Supramarginal gyrus part of Wernicke's area | 0.046 |
|  |  |  |  |  |  |
| CH32 | 71 | -46.333 | 2.667 | 21 - Middle Temporal gyrus | 0.459 |
|  |  |  |  | 22 - Superior Temporal Gyrus | 0.355 |
|  |  |  |  | 37 - Fusiform gyrus | 0.147 |
|  |  |  |  | 20 - Inferior Temporal gyrus | 0.039 |
|  |  |  |  |  |  |
| CH33 | 72 | -15.333 | 1.667 | 22 - Superior Temporal Gyrus | 0.556 |
|  |  |  |  | 21 - Middle Temporal gyrus | 0.444 |
|  |  |  |  |  |  |
| CH34 | 62.667 | 14.333 | 8.333 | 48 - Retrosubicular area | 0.414 |
|  |  |  |  | 6 - Pre-Motor and Supplementary Motor Cortex | 0.303 |
|  |  |  |  | 44 - pars opercularis, part of Broca's area | 0.228 |
|  |  |  |  | 45 - pars triangularis Broca's area | 0.052 |
|  |  |  |  | 38 - Temporopolar area | 0.003 |
|  |  |  |  |  |  |
| CH35 | 54 | 43 | 10 | 45 - pars triangularis Broca's area | 0.709 |
|  |  |  |  | 46 - Dorsolateral prefrontal cortex | 0.291 |
|  |  |  |  |  |  |
| CH36 | 28.667 | 67 | 16.667 | 10 - Frontopolar area | 0.927 |
|  |  |  |  | 46 - Dorsolateral prefrontal cortex | 0.073 |
|  |  |  |  |  |  |
| CH37 | -4.333 | 70 | 16 | 10 - Frontopolar area | 1 |
|  |  |  |  |  |  |
| CH38 | -34 | 61.333 | 17.333 | 46 - Dorsolateral prefrontal cortex | 0.522 |
|  |  |  |  | 10 - Frontopolar area | 0.478 |
|  |  |  |  |  |  |
| CH39 | -51.667 | 40.667 | 14.333 | 45 - pars triangularis Broca's area | 0.909 |
|  |  |  |  | 46 - Dorsolateral prefrontal cortex | 0.091 |
|  |  |  |  |  |  |
| CH40 | -60 | 17.333 | 14.333 | 44 - pars opercularis, part of Broca's area | 0.419 |
|  |  |  |  | 6 - Pre-Motor and Supplementary Motor Cortex | 0.268 |
|  |  |  |  | 45 - pars triangularis Broca's area | 0.217 |
|  |  |  |  | 48 - Retrosubicular area | 0.096 |
|  |  |  |  |  |  |
| CH41 | -67.667 | -10 | 11.667 | 22 - Superior Temporal Gyrus | 0.648 |
|  |  |  |  | 43 - Subcentral area | 0.217 |
|  |  |  |  | 48 - Retrosubicular area | 0.135 |
|  |  |  |  |  |  |
| CH42 | -70 | -41.333 | 8.667 | 22 - Superior Temporal Gyrus | 0.787 |
|  |  |  |  | 21 - Middle Temporal gyrus | 0.212 |
|  |  |  |  |  |  |
| CH43 | 73 | -26.667 | -11.667 | 21 - Middle Temporal gyrus | 0.853 |
|  |  |  |  | 20 - Inferior Temporal gyrus | 0.147 |
|  |  |  |  |  |  |
| CH44 | 66.333 | 1.667 | -11.333 | 21 - Middle Temporal gyrus | 0.849 |
|  |  |  |  | 38 - Temporopolar area | 0.123 |
|  |  |  |  | 48 - Retrosubicular area | 0.027 |
|  |  |  |  |  |  |
| CH45 | 57 | 34.333 | -4.667 | 45 - pars triangularis Broca's area | 0.554 |
|  |  |  |  | 38 - Temporopolar area | 0.208 |
|  |  |  |  | 47 - Inferior prefrontal gyrus | 0.147 |
|  |  |  |  | 46 - Dorsolateral prefrontal cortex | 0.091 |
|  |  |  |  |  |  |
| CH46 | 43.667 | 60.333 | -1.667 | 10 - Frontopolar area | 0.486 |
|  |  |  |  | 46 - Dorsolateral prefrontal cortex | 0.478 |
|  |  |  |  | 47 - Inferior prefrontal gyrus | 0.02 |
|  |  |  |  | 11 - Orbitofrontal area | 0.016 |
|  |  |  |  |  |  |
| CH47 | 12.333 | 74 | 2.667 | 10 - Frontopolar area | 0.848 |
|  |  |  |  | 11 - Orbitofrontal area | 0.152 |
|  |  |  |  |  |  |
| CH48 | -19.333 | 72 | 4.667 | 10 - Frontopolar area | 0.766 |
|  |  |  |  | 11 - Orbitofrontal area | 0.234 |
|  |  |  |  |  |  |
| CH49 | -44.333 | 55.667 | 3.333 | 46 - Dorsolateral prefrontal cortex | 0.759 |
|  |  |  |  | 10 - Frontopolar area | 0.241 |
|  |  |  |  |  |  |
| CH50 | -56 | 34.667 | 2.333 | 45 - pars triangularis Broca's area | 0.925 |
|  |  |  |  | 38 - Temporopolar area | 0.058 |
|  |  |  |  | 46 - Dorsolateral prefrontal cortex | 0.016 |
|  |  |  |  |  |  |
| CH51 | -62 | 3.667 | -1.333 | 48 - Retrosubicular area | 0.527 |
|  |  |  |  | 21 - Middle Temporal gyrus | 0.289 |
|  |  |  |  | 38 - Temporopolar area | 0.174 |
|  |  |  |  | 6 - Pre-Motor and Supplementary Motor Cortex | 0.01 |
|  |  |  |  |  |  |
| CH52 | -71 | -22.333 | -5.333 | 21 - Middle Temporal gyrus | 0.915 |
|  |  |  |  | 22 - Superior Temporal Gyrus | 0.085 |
